# Hepatocellular Adenoma: Evaluation with Contrast-Enhanced Ultrasound and MRI and Correlation with Pathologic and Phenotypic Classification in 26 Lesions

**DOI:** 10.1155/2012/418745

**Published:** 2012-07-02

**Authors:** Anne-Frédérique Manichon, Brigitte Bancel, Marion Durieux-Millon, Christian Ducerf, Jean-Yves Mabrut, Marie-Annick Lepogam, Agnès Rode

**Affiliations:** ^1^Department of Radiology, Croix Rousse Hospital, Grande rue de la Croix-Rousse, 69004 Lyon Cedex 04, France; ^2^Department of Pathology, Croix Rousse Hospital, 103 Grande rue de la Croix-Rousse, 69004 Lyon Cedex 04, France; ^3^Department of Digestive Surgery, Croix Rousse Hospital, 103 Grande rue de la Croix-Rousse, 69004 Lyon Cedex 04, France; ^4^Site Laccasagne, 162 Avenue Laccassagne, 69424 Lyon, France

## Abstract

*Purpose*. To review the contrast-enhanced ultrasonographic (CEUS) and magnetic resonance (MR) imaging findings in 25 patients with 26 hepatocellular adenomas (HCAs) and to compare imaging features with histopathologic results from resected specimen considering the new immunophenotypical classification. *Material and Methods*. Two abdominal radiologists reviewed retrospectively CEUS cineloops and MR images in 26 HCA. All pathological specimens were reviewed and classified into four subgroups (steatotic or HNF 1**α** mutated, inflammatory, atypical or **β**-catenin mutated, and unspecified). Inflammatory infiltrates were scored, steatosis, and telangiectasia semiquantitatively evaluated. *Results*. CEUS and MRI features are well correlated: among the 16 inflammatory HCA, 7/16 presented typical imaging features: hypersignal T2, strong arterial enhancement with a centripetal filling, persistent on delayed phase. 6 HCA were classified as steatotic with typical imaging features: a drop out signal, slight arterial enhancement, vanishing on late phase. Four HCA were classified as atypical with an HCC developed in one. Five lesions displayed important steatosis (>50%) without belonging to the HNF1**α** group. *Conclusion*. In half cases, inflammatory HCA have specific imaging features well correlated with the amount of telangiectasia and inflammatory infiltrates. An HCA with important amount of steatosis noticed on chemical shift images does not always belong to the HNF1**α** group.

## 1. Introduction

Hepatocellular adenomas (HCAs) are uncommon primary benign tumours, usually found in young and middle-aged women, typically encountered in the presence of a long history of oral contraceptive use (OCs) [1]. HCAs can be solitary and are monoclonal tumours. They are considered now as a heterogeneous entity. Several pathomolecular features have recently been described [2, 3]. Based on two molecular criteria (HNF1*α* mutations and *β*-catenin mutations) and an additional histological criterion, four subgroups can be defined: HNF1*α* mutated adenomas, *β*-catenin mutated adenomas, and inflammatory and/or telangiectatic adenomas; the fourth group has no particular morphological and molecular features. 

Each HCA subtype is potentially associated with different evolutionary risk factors. *β*-catenin mutated HCAs are more frequently associated with the development of hepatocellular carcinoma (HCC) whereas inflammatory/telangiectatic HCAs have a significant risk of haemorrhage and also a slight risk of degeneration [4].

The noninvasive differentiation of HCA from other benign tumours (especially with focal nodular hyperplasia) has remained a challenge because of their highly variable appearance. Magnetic resonance imaging (MRI) has been considered as the most comprehensive imaging workup for HCA diagnosis [5]. Contrast-enhanced ultrasound (CEUS) has been found to have a good sensitivity and specificity to characterise a focal liver lesion due to a major advantage: continuous imaging over the whole enhancement period [6]. 

We, therefore, performed a retrospective analysis of 26 HCAs in 25 patients with the aim of identifying MRI and CEUS features specifically associated with each subtype which have not been well described in the radiological literature. The originality of this study is the correlation between imaging features and pathological semiquantitative evaluation of inflammatory infiltrates, steatosis, and telangiectasias specifically according to their immunophenotypical classification.

## 2. Patients and Methods

Among the 48 HCA patients undergoing surgery at our institution hospital (from 2003 to 2010), we identified 23 patients in which liver MRI scans, CEUS, and suitable pathological materials were available. The other 25 patients were excluded from this study due to a lack of complete MRI data (*n* = 7) or contrast-enhanced US data (*n* = 18). The mean time between contrast-enhanced US and MRI was 6 weeks (0 day–9 months). Surgery was performed in a mean time of one month after imaging (0 day–7 months).

We also followed another two HCA patients with available liver biopsies, appropriate MRI and contrast-enhanced US data.

We, therefore, included a total of 25 patients (26 HCAs) in the series.

This study was conducted in agreement with French law (March 4, 2002) and the Declaration of Helsinki, and patient consent was not required.

Clinical information was collected including sex, age, circumstances of diagnosis, body mass index, oral contraceptive or anabolic use, and presence of diabetes. Biological test results such as CRP level, liver function tests, and fibrinogen were also collected. 

### 2.1. Pathologic Analysis

All cases (24 resection and 2 biopsy) were reviewed retrospectively by one pathologist (B. Bancel) with expertise in hepatic pathology. Pathological analysis was performed on paraffin-tissue sections stained with hematoxylin-eosin, Masson's trichrome, and reticulin staining. Tumour characteristics, including size, number of nodules, and presence of haemorrhage were reported. 

The extent of steatosis and sinusoidal dilatation/telangiectasia in the HCA was arbitrarily defined as a percentage of involvement at low magnification as absent (less than 5%; grade 0), mild (5% to 29%; grade 1), moderate (30% to 50%; grade 2), or severe (more than 50%; grade 3). Inflammatory infiltrates were scored asabsent (grade 0), a few inflammatory infiltrates without any foci (grade 1), one lymphocytic foci/per 10 high power fields (HPF, grade 2), or 2 and more lymphocytic foci/ per 10 HPF (grade 3). Cytologic atypia, acinar pattern, or monomorphism of the cells was recorded. In the nontumoural liver, we evaluated the fibrosis, the steatosis, and the presence of undetected nodules. If lesions were multiple, we only included nodules in which CEUS was performed and could be correlated with the resected lesion.

According to the recent classification [7], our cases were classified into four subtypes based on morphological criteria: (1) inflammatory HCA characterised by sinusoidal dilatation, abortive portal tracts, a more or less obvious ductular reaction inflammation and naked arteries; (2) steatotic HCA, lacking sinusoidal dilatation/telangiectasia and inflammation, is characterised by diffuse steatosis (>50%); (3) atypical HCA identified as those displaying some cellular monomorphism and a few acinar structures, features that by themselves are not sufficient to support a diagnosis of malignancy; (4) nodules without any of these above mentioned characteristics were reported as unclassified HCAs. 

Immunohistochemical analysis was performed on 4 *μ*m-thick formalin-fixed and paraffin-embedded sections using antibodies against liver fatty acid binding protein (LFABP Novocastra Labs, dilution 1 : 20), antiserum amyloid A (SAA Novocastra Labs, dilution 1 : 20), *β*-catenin (Novocastra Labs, dilution 1 : 20), and glutamine synthetase (Novocastra Labs, dilution 1 : 20). Full immunophenotypic characterisation was performed in only 11 cases. 

### 2.2. Imaging Protocols

All 25 patients underwent MR and CEUS before surgery.

All MR imaging examinations were performed with 1.5 T system; 12 in our hospital with Magnetom Avanto or Symphony Siemens Medical System and 13 in others imaging centres. The 12 patients underwent a preoperative MRI examination in our hospital with a dedicated phase-array coil for signal reception. The following sequences were acquired and analysed: axial breath-hold T1-weighted fast field-echo pulse sequence; axial in-phase and out-phase chemical shift GRE T1-weighted images; (repetition time/echo time 129/2,38 and 4,76 msec; flip angle, 70°; field of view, 380 mm; matrix, 158 × 256; number of sections, 30; section thickness, 6 mm; gap, 15%; two signals acquired), a respiratory-triggered, fat-suppressed, T2-weighted fast spin-echo pulse sequence (repetition time/echo time, 2100/84 msec; flip angle, 150°; field of view, 350 mm; matrix, 207 × 384; number of sections, 30; section thickness, 6 mm; gap, 10%; one signal acquired), and a fat suppressed dynamic gadolinium-enhanced T1W gradient echo sequences during the arterial phase, portal venous phase, and delayed phase, with administration of gadolinium-based contrast medium (repetition time/echo time, 3,89/1,51 msec; flip angle, 25°; field of view, 420 mm; matrix, 144 × 384; number of sections, 52; section thickness, 3 mm; gap, 20%; one signal acquired).

For the 13 patients who underwent initial MRI examination elsewhere, the inclusion criteria were the ability of at least five sequences from 1.5 T magnetic resonance machine, including in-phase and out-of-phase chemical shift GRE T1W images, fat-suppressed T2W images, and fat-suppressed gadolinium-enhanced T1W sequences during arterial, portal venous, and delayed phases.

All 25 sonographic studies were performed by one of our abdominal radiologists using Philips IU 22, Siemens Elegra or Aplio XG Toshiba. First, a baseline investigation of the liver in B-mode, using greyscales, was performed with a 3.5 MHz curvilinear-array transducer. The lesion was measured routinely. Subsequently, dynamic real-time contrast-enhanced sonography was performed using contrast-coherent imaging, with the same curvilinear-array transducer, with a focus in the area of interest. A low mechanical index (<0.2) was selected to avoid microbubble disruption. Contrast-enhanced sonographic studies were performed immediately after the administration of 2.4 mL of sulfur hexafluoride–filled micro bubbles (BR 1, SonoVue, Bracco) as a bolus with a 20 gauge peripheral IV cannula followed by 10 mL of saline and the chronometer was started. A second injection of Sonovue was performed when judged necessary or to explore a second lesion.

The entire examination was tape recorded to allow later review.

### 2.3. Imaging Analysis

**Figure 1:**
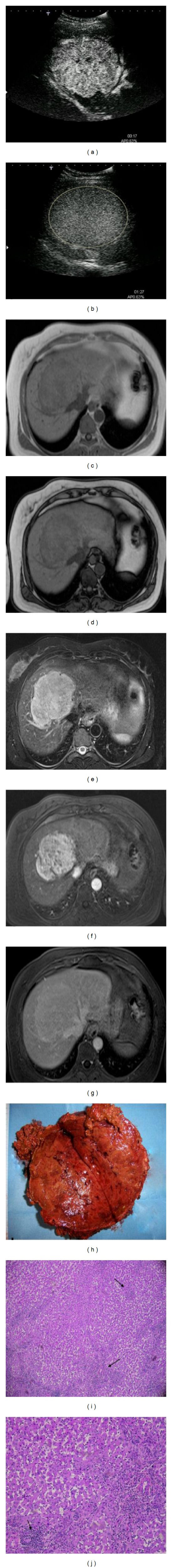
Inflammatory HCA (with typical imaging features): (a) The 10 cm lesion located in segments VIII and IV shows a marked hypervascularity after contrast injection on CEUS, (b) which is persistent in the delayed phase. (c) It is slightly hypointense with the surrounding parenchyma on T1W images with (d) no signal drop-out on the chemical shift sequence and (e) a high signal intensity on T2W fat-suppressed images. (f) It has a strong arterial enhancement after gadolinium administration, (g) which is persistent in the delayed phase. (h) Photograph of the resected specimen revealing a well-circumscribed brown mass with some vessels cut in a transverse plane. (i, j) It shows typical aspects with obvious telangiectasia (70%) and inflammatory infiltrates (grade 3) (black arrows) around thick arteries.

**Figure 2:**
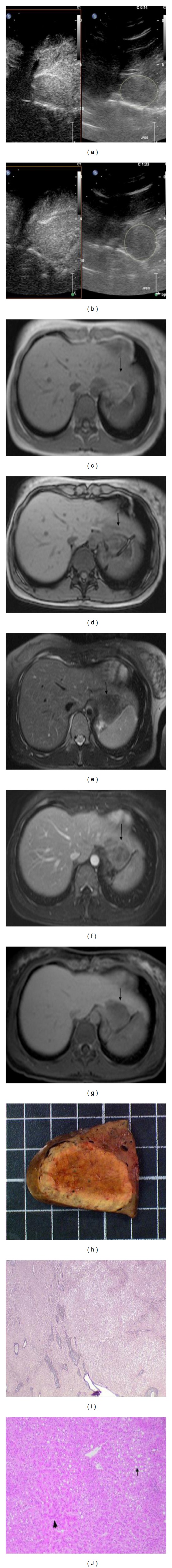
Inflammatory HCA (with less typical imaging features): (a) the 5 cm lesion has slight and homogeneous enhancement after microbubble contrast agent injection and (b) becomes isoechoic relative to the adjacent liver parenchyma. (c) The lesion (black arrow) located in segment II is isointense on T1W images with (d) a drop-out signal on chemical shift images and (e) hypointense on T2 fat-suppressed images. (f) The arterial enhancement is slight (g) with a wash out in the delayed phase. (h) Macroscopic picture: well-limited, nonencapsulated tan-colour lesion without haemorrhagic changes. (i, j) The tumour shows moderate telangiectasia (30%, arrow head) mixed with steatotic hepatocytes (40%, black arrow).

**Figure 3:**
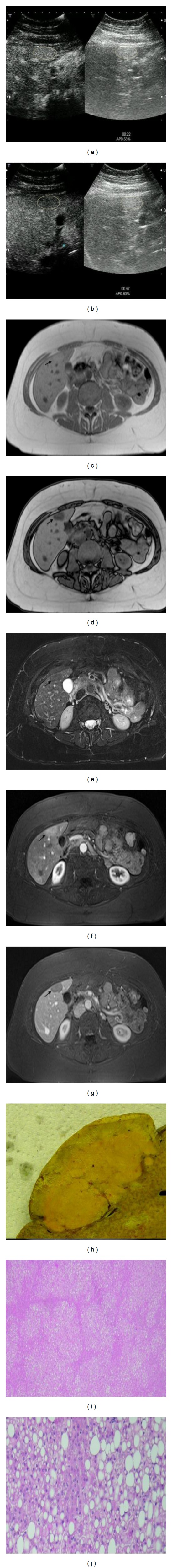
Steatotic HCA. (a) The 2.2 cm lesion, hyperechoic in B mode, has a very slight arterial enhancement, (b) which becomes isoechoic in the delayed phase after microbubble contrast agent injection. (c) The lesion (black arrow) appears isointense on T1W images with (d) an important drop-out signal on the chemical shift sequence, and (e) is hypointense on T2W images with (f) a slight arterial enhancement and (g) an apparent wash out in delayed phases. (h) Macroscopic picture shows a well limited tan-colour lesion. (i, j) Pathologic examination shows important proliferation of hepatocytes with extensive macro- and microvesicular steatosis.

All images were interpreted retrospectively and separately in consensus by two abdominal radiologists (A. Rode, M. Durieux-Millon; 17 and 7 years of experience, resp.) with knowledge of the diagnosis of HCA but without knowledge of the pathological subgroup; a consensus was obtained in cases of difference. MRI and CEUS obtained in each patient were reviewed separately (Figures 1, 2, and 3).

The U.S. patterns were analysed: in B-mode, the echogenicity (classified as hypo echoic, iso echoic, or hyper echoic to the adjacent liver parenchyma); after Sonovue injection: the enhancement pattern in arterial, portal-venous, and late phase (none, slightly, moderately or intense); the homogeneity of enhancement (homogeneous or non-homogeneous), the direction of enhancement (centripetal, centrifugal or diffuse). 

The following criteria were analysed on MRI: signal intensity of the lesion on unenhanced T1- and T2-weighted images compared to the adjacent parenchyma (hypo, iso, hyper intense); presence or not of a drop-out signal on chemical shift sequence, presence of haemorrhage within the lesion, the enhancement pattern in arterial, portal venous and late phase (none, slighlty intense, moderately intense, intense), and the homogeneity of the enhancement. 

### 2.4. Statistical Analysis

Statistical analysis was carried out using SAS software v 9.1. Qualitative variables and adenoma subtypes were compared with each other in contingency tables using a chi-square or Fisher's exact test. For quantitative variables, data were expressed as median and its minimal and maximal value. The differences between quantitative variables were evaluated with the Wilcoxon test (as a nonparametric test). All reported *P* values were 2-tailed; a *P* value of less than 0.05 was considered statistically significant. The *P* values reported in Table 2 were calculated using 3 subtypes of adenoma (inflammatory, steatotic, and atypical) and 4 subtypes (inflammatory with typical imaging features, inflammatory with less typical imaging features, steatotic and atypical). Kappa statistics were calculated to assess interobserver agreement in each assessed finding. Agreement was graduated as *κ* < 0.20, poor; 0.20–0.39, fair; 0.40–0.59, moderate; 0.60–0.79, substantial; or 0.80–1.00, almost perfect [8].

## 3. Results

### 3.1. Clinical Findings

Clinical and biological findings are resumed in Table 1.

Among the 25 patients, 23 patients were women (mean age 37 years; age range 21–52) and 2 were men (39 and 45 years old). Fifteen patients (60%) were asymptomatic and 10 (40%) had abdominal pain, with discovery of a liver haematoma in 2 (one inflammatory and one atypical HCA) cases. Sixteen patients (69.6%) had used oral contraceptives for a long period (more than two years). Three patients (12%) had type 2 diabetes (due to obesity) (with 2 inflammatory HCAs). No familial diabetes was found. Six women (24%) were overweight and had a BMI > 25, including 4 obese with a BMI > 30 (with 5 inflammatory HCAs and one with an atypical HCA). 

### 3.2. Pathological Findings

The results are summarised in Table 2.

Among the 26 nodules, 16 (62%) in 15 patients were telangiectatic/inflammatory HCAs. The extent of inflammatory infiltrate and telangiectasia varied from case to case. Telangiectasia and inflammatory infiltrate were both marked in 8 cases (50%). Steatosis was diffuse in 2, and focal in one. A haemorrhagic component was found in 2 lesions and one case had an intraparenchymal haematoma (1.3 cm). No cytological abnormalities or acinar structures were seen.

Six *HCAs* (23% of HCAs) in 6 patients were classified as *steatotic*. They displayed moderate to marked steatosis and lacked sinusoidal dilatation or inflammatory infiltrate. Steatosis was diffuse in all but one case, in which it was irregularly distributed. Two cases (diameters of 6.5 and 9 cm) had microscopic haemorrhages.

Four *HCAs* were classified as *atypical*. They did not show any inflammation. One case had a marked steatosis (50%) and one had some steatotic foci and a central haemorrhage. In one patient (3.8%), a well-differentiated HCC arose as distinct nodules within an atypical HCA (11 cm) without any vascular invasion. Demonstration of nuclear and/or cytoplasmic *β*-catenin expression was positive in only this latter case, in both areas of HCA and hepatocellular carcinoma. 

No lesion was classified in the unspecified group.

The nontumoural liver was nonfibrous, F0-1 according to Metavir in all cases.

Immunohistochemistry showed an abnormal expression of *β*-catenin and glutamine synthetase in only one case (the HCA with malignant transformation). None of the telangiectatic/inflammatory HCAs tested had an abnormal expression of *β*-catenin, but they displayed an over-expression of SAA. 

### 3.3. General Imaging Findings

All the imaging results are summarised in Table 3 (with *P* values). Substantial to almost perfect interobserver agreement (*κ* > 0.60) was achieved for all the imaging criteria.

#### 3.3.1. Inflammatory Group

Two subgroups could be distinguished on imaging: one, homogeneous, with typical imaging features (7 lesions/16 IHCA) and another one, more heterogeneous, with less typical imaging features (9 lesions/16 IHCA).

The typical imaging features observed in inflammatory HCA are as follows:

- with CEUS, a fast arterial centripetal filling and a persistent enhancement in the portal and delayed phases;
- with MRI: a strong hyper intense signal on T2W images and a strong arterial enhancement, associated with a persistent enhancement on portal and delayed phase. Six inflammatory HCAs (and none in other group) showed a hyper intense signal on T1W sequence.

In the remaining group of lesions, with less typical imaging presentation: 4 are not hyper intense on T2W sequence, 3 have a drop-out signal on chemical shift sequence. 

#### 3.3.2. Steatotic HCA

Three major MRI features were found to be significantly associated with this group.

- First the signal drop-out on chemical shift MRI sequences was found in all 6 steatotic HCAs, diffuse in 5 and focal in 1.
- Secondly, arterial enhancement was always considered to be slight or moderate.
- Finally, all lesions presented a washout on portal or delayed phase.

*The atypical group* did not have any imaging features considered to be significantly associated with it. Our case of histologically confirmed malignant transformation within an otherwise atypical adenoma (11 cm) was not suspected based on the usual radiological criteria for HCC. It appeared heterogeneous, mostly hypointense on T1W with a slight drop-out signal on chemical shift sequence, hyperintense on T2W. It showed a strong arterial enhancement on CEUS and MRI, persistent on delayed phase.

### 3.4. Correlation between Imaging Features and Pathological Findings

The two subgroups within the inflammatory group based on imaging features are correlated with pathological findings: all the HCA with typical imaging features displayed an important amount of telangiectasia (grade ≥2) associated with an inflammation infiltrates quoted ≥2 on our four grade scale; whereas in the remaining group of lesions, only one out of 9 displayed both criteria (telangiectasia grade ≥2 and inflammation ≥2), the other 8 had either important telangiectasia or marked inflammation.

The drop-out signal on chemical shift MRI sequences is well correlated with the fat repartition within the lesion. The steatotic HCA with a patchy signal drop-out on chemical shift sequences had an irregular fat repartition on pathological analysis. The five lesions with a drop-out signal on chemical shift sequence without belonging to the steatotic HCA showed an amount of steatosis ≥30%.

## 4. Discussion

The current study retrospectively investigated imaging features with MRI and CEUS, in solitary or multiple HCAs, in relation to clinicopathological characteristics, including histological subtype.

Most *inflammatory HCAs* occur in overweight or obese patients in a context of long OP use and are often associated with a biological inflammatory syndrome [9, 10]. In our study, inflammatory HCAs are characterised by their vascular changes. But in contrast to previous studies [10–12], the diagnostic circumstances of our cases were fortuitous and never due to acute bleeding, with only 2 cases of liver haematoma of less than 10 cm in diameter. A malignant transformation is also possible even in the absence of *β*-catenin mutation [9]. In this context, the radiological identification of such lesions may be useful. 

We demonstrate, for the first time, that two distinct subgroups of inflammatory HCAs can be recognised on imaging, closely correlated with severity of telangiectasias and inflammatory infiltrates. The typical imaging features, as previously described [5], are the hyperintense signal on the T2W sequence, a strong and homogeneous arterial enhancement on MRI, and persistent enhancement in the delayed phase on MRI. In addition, we find persistent enhancement in the delayed phase with CEUS confirming a good correspondence between the two imaging techniques. These typical imaging features are associated with marked telangiectasias (grade ≥2) and inflammatory infiltrates (quoted as ≥2). The other subgroup of inflammatory HCAs has a less typical imaging presentation: not always hyperintense on the T2W sequence, some cases with a wash out at delayed phase or a drop-out signal on the chemical shift sequence, with significant steatosis. These mixed (steatotic and peliotic) HCAs, according to Lewin et al. [13], should not be misinterpreted as steatotic HCAs. 

A hyperintense signal on T1W is another typical characteristic of this inflammatory group. This does not reflect sinusoidal dilatation [14] as 3 other nodules with more than 60% of telangiectasias are hypointense on the T1W sequence. The explanation of the hypersignal T1W already seen in others lesions such as some cirrhotic nodules is unknown; bleeding, fatty degeneration, and a large amount of Cu^2+^ can play a paramagnetic role in tissue and determine this hyperintensity pattern [15].

Previously, before recent pathologic and molecular studies, inflammatory HCAs were categorised with focal nodular hyperplasia. CEUS is useful to appreciate the direction of filling of the lesion and enables us to clear up the situation if a FNH is suspected. It appears that almost all of inflammatory HCA have a centripetal filling at the arterial time of injection (*P* = 0.0273), and also a persistent enhancement at the late phase. This persistent enhancement at late phase is a discriminant sign not only for focal nodular hyperplasia, but also for other HCA subgroups. 

*Steatotic HCA* is histologically considered as a homogeneous group of tumours. They are characterised by prominent steatosis without any other specific features. It is necessary to underline that these two tumourigenic pathways, HNF1-*α* inactivation, and *β*-catenin mutations, are mutually exclusive [5], so this group of steatotic HCAs is less exposed to the risk of malignant transformation. 

The homogeneity of the signal drop-out is well correlated with the diffuse repartition of fat within the lesion in pathological findings, unlike hepatocellular carcinoma, which usually has a focal steatosis. The arterial enhancement is never strong on either MRI or CEUS, and vanished in the delayed phase. The slight arterial enhancement on CEUS and MRI can be explained by a focal compression of the sinusoids due to steatosis and a less important blood flow [16]. The marked hypointensity in the MRI delayed phase is related to the slight previous enhancement and the persistent gradient of signal between the liver and the nodule due to the fat saturation. 

However, the drop-out signal on chemical shift is not specific for the steatotic HCA group: in our study, we also found 3 inflammatory HCAs and 3 atypical HCAs with drop-out signals, explained by an important amount of fat on pathologic examination. Thus, in contrast to Laumonier's study [5], a steatotic lesion, after either pathologic analysis or radiologic examination, does not systematically belong to the group of HCAs with HNF1-*α* mutations. 

*Atypical HCA* is associated with cytological abnormalities and an increased risk of malignant transformation. One out of 26 had evidence of well-differentiated HCC without vascular invasion within an atypical adenoma on pathology. The occurrence of HCC appears in different series in about 5–10% of cases [17], In a recent study [4], the risk of malignant transformation was 4% in women and 47% in men. It is observed in tumours greater than 8 cm in diameter [18]. However, the clinical relevance of *β*-catenin is poor for predicting malignant transformation as staining is focal. Only 20% of malignant HCAs in the series of Dokmak et al. [11] were mutated, a rate similar to that of Zucman-Rossi's study [19], whereas in Farges's study [4], 2/3 of HCAs with malignant transformation were *β*-catenin activated and 1/3 displayed cell atypias. 

Our patients with adenomatosis had the same final pathological classification and imaging features as the group of solitary adenomas. As discussed by previous authors [11, 20], adenomatosis is probably not a specific entity of HCA as thought until now [21], and the number does not constitute a determining factor in its evolution [22]. Multiple HCAs in the same patient belong to the same pathological group (because they are monoclonal tumours), and pathological features most “at risk” determine the final classification.

Our study has three major limitations. First, the number of patients is small. Large multi-institutional studies are needed to corroborate our findings. The frequency of complications in our study does not represent the real percentage described in the previous studies. Secondly, this is a retrospective study and blind reading was carried out in cases with a confirmed diagnosis of adenoma. Thirdly, most of our cases did not have a immunohistochemistry analysis for subtyping HCA. But the pathological features of the subgroups are well recognised, some of these immunostains are not routinely available. The pathologist's conclusion was mostly based on the morphological analysis gold standard as it has been proved in different studies [11] and because there is a good agreement between morphological analysis and immunophenotypical classification.

*In conclusion *, differentiating inflammatory HCA from FNH and other HCA subgroups is important as it significantly affects treatment decisions. Because of a significant incidence of complications (haemorrhage and malignancy), it may be useful to recognise different subgroups of HCAs. Using imaging (MRI and CEUS), some telangiectatic HCAs can be detected because of characteristic specific features: a hyperintense signal on T2W, a spontaneous hyperintense signal on T1W and a strong arterial enhancement on MRI, which is persistent in the delayed phase. This particular profile of enhancement on MRI is also well correlated with that on CEUS. 

A diffuse drop-out signal on chemical shift sequence and a slight arterial enhancement is very suggestive of a steatotic HCA corresponding to the HNF1 subgroup, but the presence of a marked steatosis is not specific to this aetiology and can also be recognised in inflammatory or atypical (corresponding to the -catenin mutated group) HCAs. 

If we are able to recognise imaging features associated with these different subgroups of HCAs, liver biopsy is probably no longer useful to confirm the diagnosis prior to resection.

## Figures and Tables

**Table 1:** Main clinical and biological data considering HCA subtype.

	Inflammatory HCA	Steatotic HCA	Atypical HCA
	patients (*n* = 15)	patients (*n* = 6)	patients (*n* = 4)
	lesions (*n* = 16)	lesions (*n* = 6)	lesions (*n* = 4)
Age median {min-max}	37 {23–50}	33,5 {26–52}	44,5 {21–47}
Sex	*F* = 14 *M* = 1	*F* = 5 *M* = 1	*F* = 4 *M* = 0
OP (or anabolic) use >2 years	10	2	4
BMI > 25 kg/m^2^	5	0	1
Diabetes	2	0	1
Inflammatory Sd (CRP or fibrinogen)	6	0	2
Cholestasis	7	0	2
Circumstances of diagnosis	by chance: 8	by chance: 5	by chance: 2
	abdominal pain: 7	abdominal pain: 1	abdominal pain: 2
Solitary HCA	8	2	2
Multiple HCA <10	2	0	1
Multiple HCA ≥10	5	4	1

OP: oestroprogestative,

BMI: body mass index,

CRP: C-reactive protein.

**Table 2:** Main pathological data considering HCA subtype.

	Inflammatory HCA *n* = 16			
	Inflammatory HCA with typical imaging features *n* = 7	Inflammatory HCA with less typical imaging features *n* = 9	Steatotic HCA *n* = 6	Atypical HCA *n* = 4
Size of the nodule analyzed {min-max} (cm)	7,6 {4,2–13}	5,5 {3,5–9}	3,75 {2,2–9}	8,25 {2,5–11}
Steatosis				
grade 0 (<5%)	4	5	0	2
grade 1 (5–29%)	3	1	0	1
grade 2 (30–49%)	0	1	0	1
grade 3 (≥50%)	0	2	6	0
Telangiectasia				
grade 0 (<5%)	0	0	6	4
grade 1 (5–29%)	0	3	0	0
grade 2 (30–49%)	2	4	0	0
grade 3 (≥50%)	5	2	0	0
Inflammatory Infiltrates				
grade 0	0	1	1	2
grade 1	0	4	5	2
grade 2	4	0	0	0
grade 3	3	4	0	0

**Table 3:** Radiological (CEUS and MRI) features considering HCA subtype.

			Inflammatory HCA (typical on imaging) *n* = 7	Inflammatory HCA (less typical on imaging) *n* = 9	Steatotic HCA *n* = 6	Atypical HCA *n* = 4	*P* values^∗^
CEUS	B mode	HypoE	3 (43%)	5 (56%)	0	1 (25%)	
		IsoE	2 (29%)	1 (11%)	1 (17%)	1 (25%)	0,2958
		HyperE	2 (29%)	3 (33%)	5 (83%) (1 heterogeneous)	2 (50%)	
	Arterial enhancement	None	0	0	1 (17%)	0	
		Slight	0	3 (33%)	2 (33%)	2 (50%)	0,1514
		Moderate	3 (43%)	3 (33%)	3 (50%)	0	
		Intense	4 (57%)	3 (33%)	0	2 (50%)	
	Delayed enhancement	Persistent	6 (86%)	0	0	2 (50%)	
		IsoE	1 (14%)	6 (67%)	3 (50%)	1 (25%)	0,0019
		HypoE	0	3 (33%)	3 (50%)	1 (25%)	
	Homogeneousness enhancement	Homogeneous	7 (100%)	5 (56%)	5 (83%)	2 (50%)	0,0517
		Heterogeneous	0	4 (44%)	1 had no enhancement	2 (50%)	
	Filling direction	Centripetal	6 (86%)	7 (78%)	1 (17%)	2 (50%)	0,1012
		Indifferent	1 (14%)	2 (22%)	4 (67%)1 had no enhancement	2 (50%)	0,0273^∗∗^

MRI	T1	HypoS	1 (14%)	3 (33%)	3 (50%)	1 (25%)	
		IsoS	4 (57%)	2 (22%)	3 (50%)	3 (75%)	0,2253
		HyperS	2 (29%)	4 (33%)	0	0	
	IN/OUT	No drop out	7 (100%)	6 (67%)	0	2 (50%)	1,79E−04
		Drop out	0	3 (44%)	6 (100%) included 1 heterogeneous	2 (50%) included 1 heterogeneous	
	T2	HypoS	7 (100%)	3 (33%)	3 (50%)	1 (25%)	
		IsoS	0	1 (11%)	1 (17%)	2 (50%)	0,1012
		HyperS	0	5 (56%)	2 (33%)	1 (25%)	
	Arterial enhancement	None	0	0	1 (17%)	1 (25%)	
		Slight	0	2 (22%)	3 (50%)	0	0,0026
		Moderate	0	2 (22%)	2 (33%)	2 (50%)	
		Intense	7 (100%)	5 (56%)	0	1 (25%)	
	Delayed phase	Persistent	6 (86%)	3 (33%)	0	2 (50%)	
		IsoS	1 (14%)	1 (11%)	0	0	0,005
		HypoS	0	5 (56%)	6 (100%)	2 (50%)	

^∗^Test made with 4 categories of HCAs (inflammatory typical on imaging, inflamm less typical on imaging, steatotic and atypic),

^∗∗^Test made with 3 categories of HCAs (inflammatory, steatotic and atypic).
